# On the Action of Bloodlettiing, Heat, Cold, and Irritants, in the Treatment of Disease

**Published:** 1869-04

**Authors:** George Johnson

**Affiliations:** Physician to King’s College Hospital; Professor of Medicine in King’s College, etc.


### ON THE ACTION OF BLOODLETTIING, HEAT, COLD, AND IRRITANTS, IN THE TREATMENT OF DISEASE.

By GEORGE JOHNSON, M.D., F.R.C.P., Physician to King's College Hospital; Professor of Medicine in King's College, etc.

The general principles which Dr. Johnson has, in this paper, endeavored to establish, are:—

1. That the object of bloodletting is to lessen hyperæmia of certain parts of the vascular system.

2. Venesection is adapted for lessening engorgements of the venous system, which is usually a result of an impeded circulation through the lungs and left heart. When there are manifest signs of engorgement of the veins and obstruction in the lungs, a feeble arterial pulse does not contraindicate venesection; and there is no inconsistency in combining the practice of venesection with the administration of stimulants.

3. Local bleeding, by leeches or by cupping, is useful in many cases of inflammation. The bleeding acts by diverting blood through the superficial arteries from the deeper arteries which supply the inflamed parts.

4. Warm baths, fomentations, poultices, and dry cupping, act in an analogous way to local bleeding, but without actually removing the blood from the system.

5. Cold contracts the vessels to which it is immediately applied. The result of this may be a sympathetic contraction of distant and deeper vessels, or a driving in of blood to deeper parts. Cold to the surface, therefore, is an uncertain remedy in cases of internal inflammation.

6. The application of strong irritants, so as to inflame the skin, in the early stage of acute internal inflammations, is a distressing, and often a mischievous, practice.

As a general rule, in cases of inflammation, those local remedies are the most efficacious which are the most painless, and which quicken in the greatest degree the cutaneous circulation. —*British Medical Journal, Nov. 7.—Ranking's Abstracts.*

